# Efficacy analysis of fiberoptic bronchoscopy combined with thoracoscopy in the treatment of mediastinal bronchogenic cysts in children

**DOI:** 10.1097/MD.0000000000043994

**Published:** 2025-08-15

**Authors:** Yujie Qian, Xiaoman Zhang, Fang Yue, Zhenlei Jia, Yun Li, Juan Du, Zhiguo Chen, Ling Zhao, Guangxin Tuo, Xiangqian Ji

**Affiliations:** a Department of Thoracic Surgery, Hebei Children’s Hospital Affiliated to Hebei Medical University, Shijiazhuang, Hebei, PR China; b Department of Thoracic Surgery, The Fifth People’s Hospital of Hebei Province, Shijiazhuang, Hebei, PR China; c Hebei Children’s Health and Disease Clinical Medical Research Center, Shijiazhuang, Hebei, PR China.

**Keywords:** bronchogenic cysts, fiberoptic bronchoscopy, pediatric mediastinal tumors, thoracoscopy

## Abstract

This study explores the clinical efficacy of perioperative application of fiberoptic bronchoscopy (FB) combined with thoracoscopic minimally invasive surgery for mediastinal bronchogenic cysts in children. A retrospective analysis was conducted on 48 pediatric patients with mediastinal tumors who were admitted to our hospital from January 2015 to December 2023. Postoperative pathological results confirmed the diagnosis of bronchogenic cysts. The patients, aged 1 to 8 years (mean 4.8 ± 1.59 years), included 27 males and 21 females. Group A (25 cases) underwent preoperative and postoperative FB exploration and lavage, along with thoracoscopic cyst resection. Group B (23 cases) received thoracoscopic cyst resection alone. The preoperative general conditions, operative time, intraoperative blood loss, tube removal time, drainage volume, and other indicators were compared between the 2 groups. All children successfully underwent surgery without mortality or recurrence. There were no significant differences in age, gender, surgical time, or blood loss between the 2 groups (*P* > .05). However, Group A had significantly shorter postoperative chest drainage time, lower drainage volume, and shorter hospital stay compared to Group B. Group A had only 1 case of pneumonia and 2 cases of pleural effusion, while Group B had 4 cases of pneumonia and 3 cases of pleural effusion. The overall complication rate was lower in Group A (8.6%) than in Group B (30.4%). Thoracoscopy is effective for cyst removal, and the application of FB during the perioperative period can significantly reduce postoperative complications, shorten recovery time, and improve the quality of life in pediatric patients.

## 1. Introduction

Mediastinal tumors are common thoracic diseases with diverse histological origins and largely unclear pathogenesis.^[1,2]^ Bronchogenic cysts (BC), congenital mediastinal tumors caused by abnormal development of the tracheobronchial tree during embryogenesis, account for 10% to 15% of pediatric mediastinal tumors. Although most cysts are benign and asymptomatic,^[3]^ symptomatic cases may present with cough, dyspnea, dysphagia, or even malignant transformation, posing life-threatening risks.^[1,4]^ Thus, timely surgical intervention is crucial.^[5]^ Surgical resection remains the gold standard.^[6]^ However, optimizing surgical approaches for pediatric patients to enhance efficacy and minimize complications requires further exploration.

Traditional open thoracotomy, associated with significant trauma and prolonged recovery, has limited clinical utility.^[1,7]^ With advancements in minimally invasive techniques, thoracoscopy has become the preferred method for mediastinal tumor resection due to its reduced trauma and faster recovery.^[8–10]^ However, standalone thoracoscopy may still entail prolonged postoperative drainage and complications for BC. Ortiz et al^[11]^ analyzed 45 pediatric patients with BC, among whom 90% underwent thoracoscopic surgery. Postoperatively, 7 cases (15%) developed complications. The authors concluded that thoracoscopic resection is a feasible option for BC. However, standalone thoracoscopy has limitations in assessing airway secretions and anatomical relationships with adjacent structures, necessitating adjunct techniques to further reduce postoperative complications.

Fiberoptic bronchoscopy (FB), as an important diagnostic and therapeutic tool, allows direct visualization of the airway and clearance of secretions, reducing the risk of infection. It can also provide a detailed assessment of the relationship between the cyst and the airway.^[12,13]^ Therefore, combining FB with thoracoscopy in the treatment of mediastinal BC may offer significant advantages in reducing postoperative complications, precise lesion localization, and accelerating recovery. Wang et al^[14]^ performed perioperative evaluations using FB in 15 patients with plastic bronchitis. With a maximum follow-up of 7 years, none of the children experienced recurrence of plastic bronchitis or chronic cough.

Children, as the main patient group for mediastinal BC, face higher treatment difficulty and complexity. Due to their immature physical development, children have higher surgical risks and weaker postoperative recovery abilities compared to adults. Therefore, how to maximize the reduction of trauma to pediatric patients while ensuring efficacy is an important research goal.

The purpose of this study was to explore the clinical efficacy of perioperative FB combined with thoracoscopic minimally invasive surgery in the treatment of congenital mediastinal BC in children. By retrospectively analyzing the clinical data of 48 pediatric patients admitted to our hospital from January 2015 to December 2023, and comparing the preoperative, intraoperative, and postoperative clinical data between Group A (FB combined with thoracoscopy group) and Group B (thoracoscopy group), this study aimed to provide a safer and more effective treatment option for congenital mediastinal BC in children, further promoting the development and progress of pediatric thoracic surgery.

## 2. Materials and methods

We reviewed 48 consecutive pediatric patients who underwent the FB combined with thoracoscopy or thoracoscopy for BC treatment at Children’s Hospital of Hebei Province from January 2015 to December 2023. The institutional review board approved the study, and all parents of the young child patients or guardian provided informed consent for study inclusion.

Inclusion criteria: Pediatric patients aged 14 years or younger. Histologically confirmed bronchogenic cyst postoperatively. Complete clinical data. Parental or guardian consent for surgery and postoperative follow-up. No other medical history and able to tolerate surgery.

Exclusion criteria: Patients older than 14 years. Recurrent cases requiring second surgery. Coexisting conditions such as congenital heart disease, immunodeficiency, genetic metabolic diseases, severe chest wall deformities, etc, that significantly affect surgery. Conversion to thoracotomy during surgery for various reasons. Abandonment of treatment or death during the course.

According to the inclusion and exclusion criteria, this study enrolled 48 pediatric patients with postoperative pathological confirmation of BC. The patients ranged in age from 1 to 8 years, with a mean age of 4.8 ± 1.59 years. There were 27 males and 21 females. Before treatment, the advantages and disadvantages of BC, as well as the associated costs, were thoroughly explained to the parents or guardians of the children. Based on parental consent, the children were divided into 2 groups. Group A included 25 children (15 males and 10 females, with a mean age of 4.6 ± 1.6 years), all of whom underwent preoperative and postoperative FB for exploration and lavage, with cyst removal performed via thoracoscopy during surgery. Group B included 23 children (12 males and 11 females, with a mean age of 5.1 ± 1.5 years), who underwent cyst removal solely through thoracoscopy. Among the 48 patients in this study, 25 children were asymptomatic and were diagnosed with mediastinal tumors after presenting with pneumonia and undergoing chest CT scans. 9 children experienced dysphagia, 8 had cough with wheezing, 3 had dyspnea, 2 had sternum pain, and 1 had facial edema.

## 3. Clinical evaluation

Detailed records were kept of the children’s age, gender, onset of illness, weight, and other general conditions. Preoperative and postoperative chest CT scans were compared. The surgical approach, surgical time, intraoperative blood loss, chest drainage time, total drainage volume, postoperative inflammatory markers (white blood cell [WBC] count, C-reactive protein), postoperative complication rates, and postoperative hospital stay were recorded for all patients.

## 4. Preoperative FB procedure

**Figure 1.**
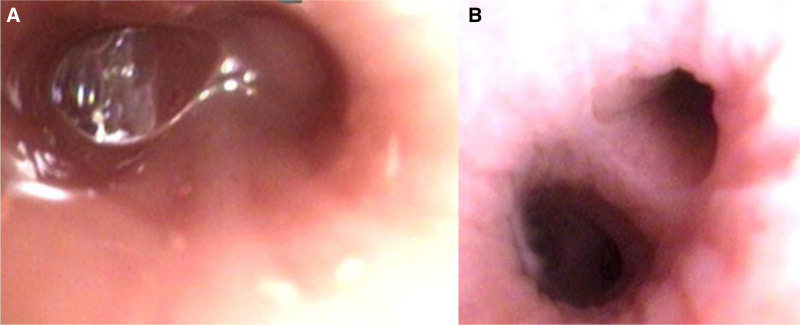
This is a patient who underwent preoperative bronchoscopy lavage. (A) A large amount of secretion can be seen through fiberoptic bronchoscopy before surgery. (B) After bronchoscopy lavage, the secretion is significantly reduced compared to before.

The FB was performed 1 to 2 days before surgery. The patient was placed in the supine position. After successful intravenous anesthesia, the fiberoptic bronchoscope was inserted into the nasal cavity. During the procedure, normal vocal cord movement was observed. The tracheal mucosa appeared normal, with no redness, swelling, or malformations. If yellowish-white sputum was present, it was aspirated. The main and branch bronchi of both lungs were examined for normal position and morphology, and any signs of mucosal congestion or swelling were noted. If white mucus was present, it was lavaged with 37°C normal saline. Based on the lesion location indicated by chest CT, lavage was performed with 37°C normal saline (1 mL/kg per lavage, ≤20 mL/lavage, total volume ≤ 5–10 mL/kg). The lavage fluid was then aspirated using a negative pressure of 100 to 200 mm Hg (1 mm Hg = 0.133 kPa), ensuring that the bronchial cavity did not collapse during aspiration. The reabsorption rate of the lavage fluid should be ≥ 40%. The anatomical relationship between the mediastinal tumor and surrounding tissues was also assessed to determine the presence of key vessels. After the procedure, the bronchoscope was withdrawn, and the lavage fluid was sent for examination. Budesonide was retained locally. Antibiotic and mucolytic treatments were continued, and the child was advised to resume a liquid diet after 4 hours, with close monitoring of their condition (Fig. 1).

## 5. Surgical procedure

**Figure 2.**
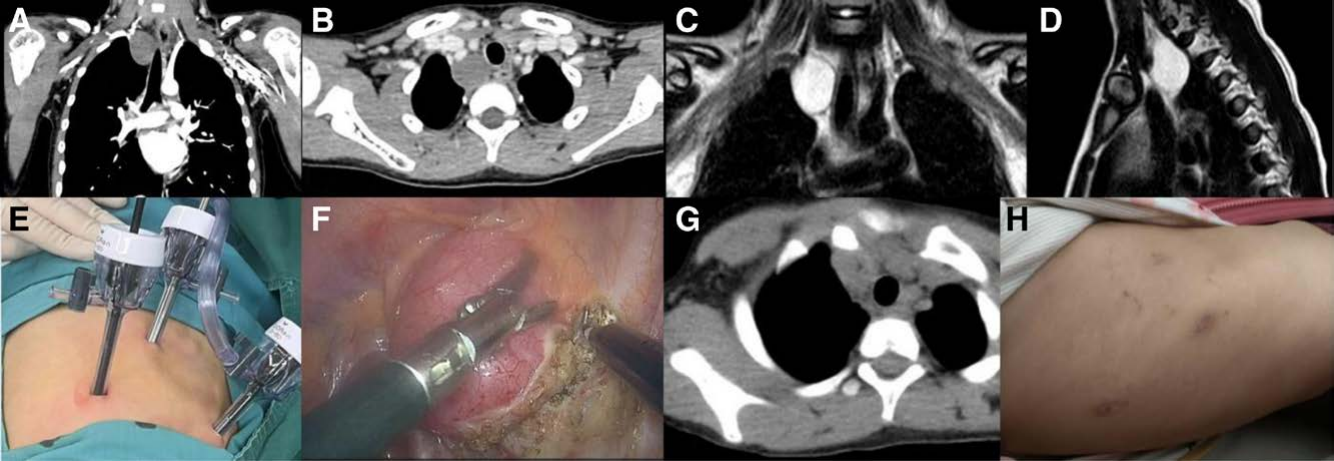
This is a 7.5-yr-old patient who was diagnosed with pneumonia through a chest CT scan. (A–D) The cysts can be seen to have caused compression of the surrounding tissues. Therefore, the parents insisted on surgery. (E) The surgical incision and the location of the puncture card can be seen in the image. (F) The cyst was removed during the operation. (G) Postoperative chest CT showed that the cyst had been completely removed. (H) The surgical wound healed well.

After satisfactory anesthesia, the patient was positioned in the left lateral decubitus position. The skin was routinely disinfected, and sterile surgical drapes were applied. A straight incision of approximately 0.7 cm was made at the fifth intercostal space along the posterior axillary line on the right side, and a thoracoscope was inserted. Under direct vision, additional incisions of approximately 0.7 cm were made at the fourth intercostal space along the anterior axillary line and the ninth intercostal space along the posterior axillary line, and trocars were inserted. Exploration revealed a round mass of approximately 3 cm × 3 cm × 2 cm at the right posterior mediastinal pleural apex. The base of the mass was located at the pleural apex, adjacent to the superior vena cava and subclavian artery. The mass was dissected in an avascular area and completely resected. After thorough hemostasis, the tumor tissue was removed in pieces through the fourth intercostal space along the anterior axillary line. The tumor was cystic. The pleural cavity was rinsed repeatedly to ensure no tissue remnants or active bleeding. After lung inflation, satisfactory lung expansion was observed. A chest drainage tube was placed at the fifth intercostal space along the posterior axillary line. The surgical instruments were counted, and the muscle and skin layers were sutured with absorbable sutures. The surgical incision was dressed with sterile dressing (Fig. 2).

## 6. Postoperative management

On the first postoperative day, a chest X-ray was performed to assess the position of the drainage tube and lung expansion. The nurse was required to record the color and volume of the drainage fluid daily, replace the drainage bottle, and observe for any fluctuations in the drainage fluid. The nurse also assisted the patient in coughing by tapping on their back and blowing up a balloon to promote lung expansion. The drainage tube was removed based on the condition of the drainage fluid, typically when the drainage volume was <50 mL for 3 consecutive days, the drainage color was normal, and the chest X-ray was normal. On the third postoperative day, a postoperative FB was performed based on the patient’s general condition. Dressing changes were conducted every 3 days. Before discharge, a chest CT or MRI was performed to assess the resection of the mediastinum tumor and confirm the absence of abnormalities in the chest.

## 7. Statistical analysis

SPSS 26.0 (IBM, Armonk) software was used to statistically analyze the preoperative and postoperative data of patients. Continuous data were presented with mean ± standard deviation (X ± SD) and their normality status was detected by Kolmogorov–Smirnov test, whereby Student *t* test was used for between-group comparisons, paired Student *t* test for in-group comparisons. Categorical data were presented with number and percentages, and Chi-square or Fisher’s exact test was used for between-group comparisons. *P* < .05 was considered as statistically significant for all analyses.

## 8. Results

All children successfully completed the surgery without any mortality or severe complications. There were no significant differences in age, gender, weight, admission temperature, or clinical symptoms between the 2 groups at admission (*P* > .05). Comparison of preoperative and postoperative imaging results in both groups revealed that the mediastinal tumors were completely resected, and the postoperative pathological results confirmed mediastinal BC. No recurrence was observed at the last follow-up (Table 1).

**Table 1:** Comparison of perioperative data between Group A and Group B.

Variabe	Group A	Group B	*P*
Age (yr)	4.6 ± 1.6	5.1 ± 1.5	.28
Male/female	15/10	12/11	.59
Admission temperature	37.2 ± 0.6	37.2 ± 0.5	.90
Weight (kg)	18.0 ± 3.9	18.7 ± 3.4	.47
Clinical symptoms			.92
Dysphagia	5 (20.0%)	4 (17.4%)	
Cough with wheezing	4 (16.0%)	4 (17.4%)	
Dyspnea	2 (8.0%)	1 (4.3%)	
Posterior sternal pain	1 (4.0%)	1 (4.3%)	
Facial edema	1 (4.0%)	0 (0.00%)	
Pneumonia	12 (48.0%)	13 (56.5%)	
Duration	9 (5,16.5)	7 (4,10)	.20
Operative time (min)	45(40, 50)	58(39, 70)	.14
Intraoperative blood loss (mL)	9(5, 15)	9(5, 15)	1
Drainage tube removal time (d)	3(3, 3.5)	5(5, 6)	<.001
Drainage volume (mL)	85(68.5, 148)	275(235, 345)	<.001
Postoperative mean body temperature (℃)	36.9 ± 0.2	37.0 ± 0.2	.14
WBC (×10^9^/L)	11.6 ± 3.2	11.9 ± 4.0	.76
CRP (mg/L)	10.0 ± 3.9	11.9 ± 3.1	.76
Hospitalization time (d)	8(8, 8)	12(10, 13)	<.001

CRP = C-reactive protein, WBC = white blood cell.

In Group A (FB combined with thoracoscopy), 2 patients developed pleural effusion and 1 patient had pulmonary inflammatory changes postoperatively. In Group B (thoracoscopy only), 3 patients developed pleural effusion and 4 developed pneumonia postoperatively. All complications improved after active treatment. A total of 3 patients (8.6%) in Group A and 7 patients (30.4%) in Group B experienced complications. Although these were not severe, a higher proportion of pulmonary symptoms were observed in Group B. This suggests that the proportion of patients with pneumonia was significantly lower in Group A after FB.

There were no statistically significant differences between the 2 groups in terms of operative time, intraoperative blood loss, postoperative mean body temperature, WBC count, and C-reactive protein levels (*P* > .05). However, the longest operative time in Group B was 95 minutes and the shortest was 35 minutes, compared to 85 minutes and 30 minutes in Group A, respectively, indicating a slight advantage for Group A.

The median time for chest drainage tube removal in Group A was 3 days, compared to 5 days in Group B, with a statistically significant difference (*P* < .001). The median drainage volume in Group A was 85 mL, compared to 275 mL in Group B, with a statistically significant difference (*P* < .001). The median hospital stay was 8 days in Group A and 12 days in Group B, with a statistically significant difference (*P* < .001; Table 1).

## 9. Discussion

This study retrospectively analyzed the clinical data of 48 pediatric patients with mediastinal BC in our hospital, comparing the therapeutic differences between Group A (FB combined with thoracoscopy) and Group B (thoracoscopy alone). The results showed that Group A had significant advantages in reducing postoperative complications, chest drainage time, total drainage volume, and hospital stay compared to Group B. The study demonstrated that the combined application of FB and thoracoscopy during the perioperative period can optimize the surgical process, reduce surgical risks, and promote patient recovery, holding significant clinical importance.

Currently, thoracoscopic surgery has been widely established as the preferred treatment for mediastinal cysts.^[7,8]^ However, standalone thoracoscopic procedures are associated with relatively high rates of postoperative complications, such as pneumonia and pleural effusion. In this study, the combined application of FB significantly reduced complication rates from 30.4% (Group B) to 8.6% (Group A), demonstrating marked prognostic improvement. The results indicating that this technology can significantly improve prognosis through the following mechanisms: Precise preoperative assessment and pretreatment: FB allows for direct visualization of the airways, removal of secretions, and lavage, reducing the risk of infection.^[13]^ In this study, 25 patients in Group A underwent lavage before surgery, which could reduce airway bacterial load and the risk of postoperative pneumonia (4% in Group A vs 17.4% in Group B). Additionally, FB can clarify the anatomical relationship between the cyst and airways and blood vessels, allowing for preoperative surgical planning, assisting in precise intraoperative dissection, avoiding injury to critical structures, reducing intraoperative time, and improving patient prognosis. Proactive intervention for postoperative complications: Postoperative application of FB can detect and promptly manage airway edema or mucus retention, facilitating lung re-expansion and thereby shortening chest drainage time (significantly lower average drainage time in Group A than in Group B).^[14]^ This advantage is particularly important in pediatric populations, as children have narrower airways and relatively weaker secretion clearance ability compared to adults, so postoperative inflammatory responses can exacerbate respiratory symptoms.

The clinical manifestations of pediatric mediastinal BC are often concealed, with most patients presenting with respiratory symptoms,^[15]^ and the cysts are mostly located in the middle and posterior mediastinum^[16]^ (accounting for 80% in this study), which can easily compress the trachea or esophagus, causing dyspnea or dysphagia.^[4]^ In this study, Group A applied FB combined with thoracoscopic minimally invasive technology in the perioperative period, not only shortening the length of hospital stay but also significantly reducing postoperative complications, confirming the adaptability of the “dual minimally invasive” strategy to pediatric physiological characteristics. Additionally, FB lavage fluid can be sent for preoperative examination, allowing for early etiological diagnosis based on pathogenic results and guiding antibiotic use, thereby avoiding excessive antibiotic use.^[17]^ Furthermore, this study expands the scope of perioperative management by incorporating FB. Compared with thoracoscopy alone,^[7]^ our combined approach reduced drainage duration from 5 days to 3 days and decreased drainage volume by 69% (85 vs 275 mL). These results collectively demonstrate the unique advantages of this combined technique for pediatric populations.

This study was a single-center retrospective study with a small sample size (48 cases), which may introduce selection bias. Additionally, the grouping was based on parental willingness rather than a randomized controlled design, which may affect the objectivity of the results. Future studies should conduct multicenter, large-sample randomized controlled trials and extend follow-up periods to assess long-term efficacy (e.g., cyst recurrence rate). Moreover, FB requires high anesthesia and operator technical skills, and its promotion should be combined with a cost-effectiveness analysis of medical resource distribution.

## 10. Conclusion

The combined application of FB and thoracoscopy in the treatment of pediatric mediastinal BC, through precise perioperative assessment and intervention, significantly reduces complications and accelerates recovery. This approach exemplifies the integration of minimally invasive surgery and refined management. This strategy offers new insights into the development of pediatric thoracic surgical techniques. Future validation of its long-term benefits and applicability will require higher-quality studies.

## Author contributions

**Conceptualization:** Yujie Qian, Fang Yue, Zhiguo Chen, Ling Zhao, Xiangqian Ji.

**Data curation:** Yujie Qian, Xiangqian Ji.

**Formal analysis:** Yujie Qian, Fang Yue, Yun Li, Juan Du, Xiangqian Ji.

**Investigation:** Yujie Qian, Zhenlei Jia, Yun Li, Juan Du, Xiangqian Ji.

**Methodology:** Yujie Qian, Xiaoman Zhang, Fang Yue, Zhenlei Jia, Yun Li, Zhiguo Chen.

**Project administration:** Xiaoman Zhang, Zhiguo Chen.

**Resources:** Yujie Qian, Xiaoman Zhang, Fang Yue, Yun Li, Juan Du, Ling Zhao, Xiangqian Ji.

**Software:** Xiaoman Zhang, Ling Zhao, Xiangqian Ji.

**Supervision:** Guangxin Tuo, Xiangqian Ji.

**Validation:** Xiangqian Ji.

**Visualization:** Xiaoman Zhang, Zhenlei Jia, Guangxin Tuo.

**Writing – original draft:** Yujie Qian, Xiaoman Zhang, Xiangqian Ji.

**Writing – review & editing:** Yujie Qian, Xiaoman Zhang, Xiangqian Ji.
